# Enhancing the Design of Microdevices: The Role of Computational Fluid Dynamics and Experimental Investigation

**DOI:** 10.3390/mi16030316

**Published:** 2025-03-09

**Authors:** Behrouz Pirouz, Hana Javadi Nejad, Anna Selene Chirillo, Seyed Navid Naghib, Patrizia Piro

**Affiliations:** 1 Department of Civil Engineering, University of Calabria, 87036 Rende, Italy; hana.javadi@unical.it (H.J.N.); navid.naghib@unical.it (S.N.N.); patrizia.piro@unical.it (P.P.); 2 ASP Cosenza, 87100 Cosenza, Italy; selenechirillo@gmail.com

**Keywords:** microfluid, medical sensors, microdevices, capillary flow, CFD

## Abstract

The growing use of microfluidic-based devices necessitates an analysis of flow characteristics through both experimental methods and computational fluid dynamic (CFD) simulations. CFD simulations facilitate the investigation of various devices, including medical sensors, by providing detailed insights into flow behavior. In this study, we conducted experimental and CFD analysis of the microfluidic flow in three devices: a COVID-19 rapid test kit, a blood glucose kit, and a PDMS kit. Our findings revealed that the changes in wall adhesion (contact angles) during the capillary flow could cause significant deviation from theoretical flow speed predictions. A hemodynamic analysis of the blood glucose kit and PDMS kit showed that capillary filling decreased in length, and flow speed could depend on the microchannel diameter. CFD results indicated the prominent role of porosity in the simulation of porous media material such as the COVID-19 test kit, as well as surface tension coefficients and wall adhesion (contact angles) in blood glucose kits and PDMS kits. Therefore, considering adaptive dynamic contact angles in CFD simulation software such as Ansys-Fluent 2024 could result in a more accurate prediction than simplified theoretical techniques, which is useful for sensor optimization and development.

## 1. Introduction

Today, there are many microfluid-based devices, such as smart textiles [1], Lab-on-a-Chip (LoC) [2], inkjet printers [3], microfabrication [4], and so on. There are different types of LoC devices to identify diseases, including those developed on a paper or polymeric platform or those manufactured including microchannels [5]. Microfluidics focuses on regulating and controlling fluids in a single or a network of microchannels at a micro-scale [6]. Major types of microfluidic systems include continuous-flow microfluidic chips, microchip electrophoresis systems, droplet microfluidics, digital microfluidic systems, Centrifugal microfluidic chips, and paper microfluidic chips [7]. Moreover, the flows are generally incompressible, viscous, and in laminar condition [8]. The generation of turbulence flow in microchannels is complex, but it is still possible with a high-pressure head and pressure difference at two points [9,10]. In a microfluidic devices’ fluid flow mechanism, multiple physical effects are involved, such as pressure gradients, electrokinetics, and capillarity [11]. Depending on the application of the microdevices, these parameters usually need to be controlled by passive or active control techniques to achieve the desired fluid flow time and velocity to detect and analyze the used liquid [12]. The fluid flow in micro devices often involve complex fluid dynamics that require consideration of proper analysis approach depending on the dimensions and characteristics of the microdevice [13].

In the macro-scale, the two analysis approaches are Lagrangian (tracking the fluid path by fluid behavior such as velocity and position during the time) and Eulerian (examining the changes in fixed points properties like velocity over time) [14]. However, in micro-scales, the analysis approaches include Direct Simulation Monte Carlo (DSMC), Molecular Dynamics (MD), Dissipative particle dynamics method (DPD), and Lattice Boltzmann methods (LBM) [15]. In LBM, the simulation is based on fluid density on a lattice and not Navier–Stokes equations [16]. It can be noted that, in some cases, such as in microchannels, the fluids can be considered a continuum and may be simulated by Navier–Stokes equations as the intermolecular distance in fluids is about 0.3 nm, negligible compared to the mm scale of microchannels [17]. Moreover, the flow distribution in microchannels is often driven by capillary forces that can be affected strongly by the channel shape [18].

Many researchers investigated the microdevices through experimental and simulation tools. Zhang et al. [19] analyzed the multiphase model’s role in the microdevices simulation. Their outcomes exhibited promising modeling applications in analyzing the impact of flow dynamics, including inlet injection, particle concentration, and flow velocity. Hodgson et al. [20] investigated the correlations among the simulation and experiment data of the contact angles as an important parameter in capillary flow, considering hydrophobic and hydrophilic surfaces. In another study, Xue et al. [21] did a parametrical study on the capillary flow in the parallel microchannels, and the maximum suction distance was validated by experimental data. One simulation method for the analysis of the microdevices is CFD. Carvalho et al. [22] investigated the analysis of microfluidic devices, including mass transfer in microchannels and porous zones. Moreover, the application of CFD simulation and its results revealed the challenges and advantages. In another study, Tonomura et al. [18] used CFD simulation to design a plate-fin microdevice. The results showed that the outlet manifolds can be extended to have a uniform flow, and microchannels with longer branches can be used. Furthermore, Ahmed et al. [23] analyzed microfluidic behaviors using experimental analysis and CFD simulation by Ansys-Fluent. Their results showed the impacts of external atmospheric features on the output. In addition, Llano-Serna et al. [24] used CFD to design a liquid membrane in a Taylor flow (LMTF) system for extracting lactic acid and a drop separation device to enhance downstream processing in bioprocesses. They demonstrated that CFD simulations can used for evaluating geometric flexibility, accelerating prototyping, and minimizing the need for initial experiments. The study of Garcia et al. [25] determined the uncertainty in microfluidic CFD simulation. Their results showed that the geometrical uncertainties in the microscale could significantly affect the simulation outcomes. Frolov et al. [26] investigated hemodynamics in an internal carotid artery using CFD and MRI (magnetic resonance imaging) methods and flow changes after the placement of a flow diverter (FD) in the intra-aneurysmal flow. Their results determined that the application of CFD in predicting the results of FD treatment could be helpful. Cito et al. [27] used Ansys-Fluent and volume of fluid (VOF) models with adaptive mesh for numerical and experimental investigations of uniform fluid distribution for droplet formation in parallelized microfluidics. In similar studies, Schönfeld and Hardt [28] applied the VOF model and Ansys-Fluent to investigate dynamic contact angles in CFD simulations, but they are not considered for mesh independence and model validation.

The previous studies showed the differences between the macro-scale and micro-scale and the approaches for analyzing and simulating flow in both scales. Moreover, it is determined that the performance and simulation results of CFD simulations in complex fluid flow and micro scales could be appropriate [29,30,31], such as the optimal design of the microdevices [32], bio-CFD, design of the LoC devices [33], microfabricated hyperbolic contractions analysis [34], pathogen transmission modeling [35], and optimization of the shape, dimensions, and flow uniformity [18]. However, the differences in the simulation of various medical microdevices, including porous zones, microchannels, and different human body fluids, are missed in the previous studies. Therefore, this study aims to analyze capillary-driven flow in three medical microdevices using experimental and computational fluid dynamic (CFD) simulation. Moreover, this research investigates the application of CFD in the simulation of medical micro-devices/sensors involving biological fluids along with its boundary conditions (in the microporous zone and microchannel) and validation procedures, simplifications, uncertainties, optimization methods, and advantages. The results would provide useful information about the microfluidic flow behavior of bio-samples in the microporous zone of the COVID-19 rapid test kits, hemodynamics in microchannels of blood glucose kits and microchannels of the PDMS kits, the impact of kit length and thickness, and prominent factors in CFD simulations of human body fluids within microdevices.

## 2. Materials and Methods

All methods in this study were carried out in accordance with relevant guidelines and regulations.

Three case studies are as follows:

- Case 1: Experimental and CFD investigation of bio-sample in a COVID-19 kit;
- Case 2: Experimental and CFD analysis of hemodynamics in a blood glucose kit;
- Case 3: Experimental and CFD analysis of hemodynamics in a PDMS kit (Polydimethylsiloxane).

### 2.1. Experimental Setup

**Figure 1:**
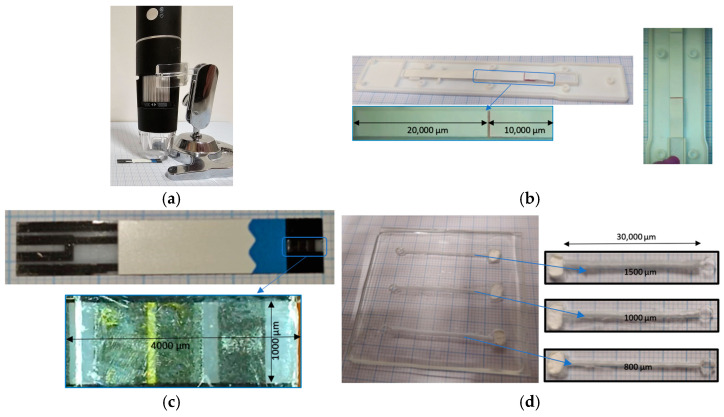
Used devices and dimensions of sensors: (**a**) digital microscope with HD capture snapshots; (**b**) used COVID-19 rapid test kit and its dimensions; (**c**) used blood glucose kit and its dimensions; and (**d**) used microfluidic PDMS with three widths of microchannels and its dimensions.

Figure 1 presents the used digital microscope (Figure 1a), microdevices, and their dimensions (Figure 1b–d). The experimental data were gathered using a digital microscope (50 to 1000×) with HD capture snapshots (100 frames per second (FPC), which means 100 still images each second). The analysis in the three case studies is on a micro-scale, not a nanoscale, and the flow speeds are milliseconds. Therefore, the selected device was applicable for data logging.

The experimental analysis of bio-sample capillaries in the porous zone has been performed on a COVID-19 rapid test kit with a thickness of 200 μm, width of 3000 μm, and length of 20,000 μm, Figure 1b. A blood glucose kit with a width of 1000 μm, length of 4000 μm, and thickness of 200 μm was used to analyze the hemodynamics, Figure 1c. Moreover, a PDMS with three sizes of 800 μm, 1000 μm, and 1500 μm has been selected to compare the flow speed in different microchannels, as shown in Figure 1d. As can be seen in the figure, the length and depth of all three microchannels are the same and equal to 30,000 μm and 1000 μm, respectively. The experimental errors in the measurements of three microdevices are presented in Table 1.

### 2.2. Numerical CFD Model Setup

The numerical model setup contains three steps, including 3D model development, meshing, and defining boundary conditions.

#### 2.2.1. Three-Dimensional Modeling

**Figure 2:**
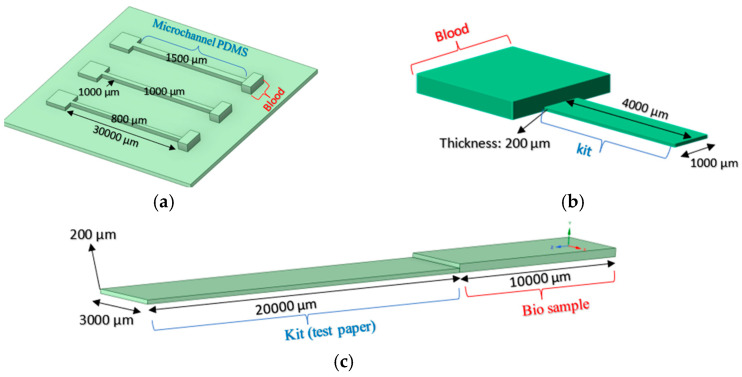
Three-dimensional model of selected micro devices: (**a**) PDMS with three widths of microchannels; (**b**) blood glucose kit; and (**c**) COVID-19 rapid test kit.

The 3D models of the COVID-19 rapid test kit, blood glucose kit, and PDMS kit have been developed using Ansys-SpaceClaim 2023 and are shown in Figure 2.

#### 2.2.2. Mesh Details and Validation

**Figure 3:**
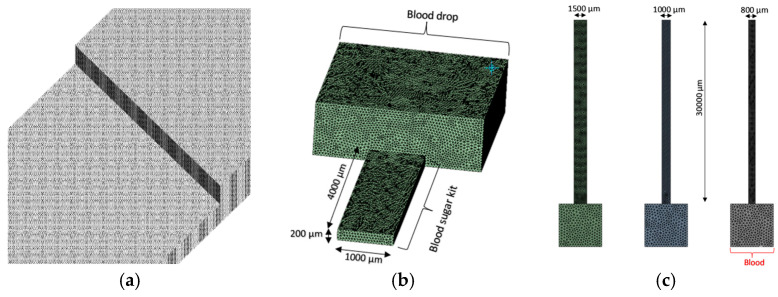
Mesh details of the microdevices: (**a**) COVID-19 rapid test kit; (**b**) blood glucose kit; and (**c**) PDMS kit.

**Figure 4:**
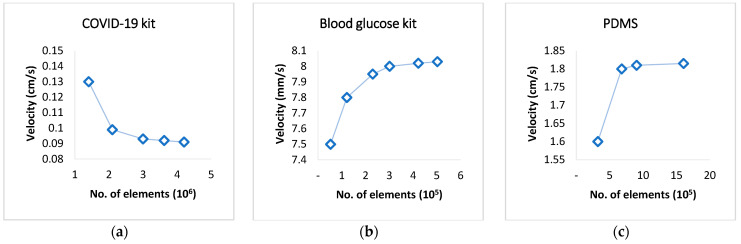
The grid independence analysis for three devices: (**a**) COVID-19 rapid test kit; (**b**) blood glucose kit; and (**c**) PDMS kit.

The mesh details of the three case studies are presented in Table 2 and Figure 3, and the grid independence analysis for the three micro devices is shown in Figure 4.

#### 2.2.3. Boundary Condition

The material properties and boundary conditions are presented in Table 3 and Table 4.

One important parameter is the viscosities of the materials, that is, the fluid’s rate-dependent resistance, which is presented in Table 3. The bio-samples’ viscosity is reported according to the applied COVID-19 rapid test kit, and the value is presented in Table 3. Blood is a complex non-Newtonian (viscoelastic) fluid, and its viscosity is not constant, changing based on stress (force). Moreover, red blood cell (RBC) deformation exists during movement. However, the dynamical deformation can be ignored for simulation on a micro-scale, such as a microchannel [36], and the Newtonian fluid model could be applicable [37].

Another factor is porosity, also known as the void fraction, which measures the void spaces in a material and ranges from 0% to 100%. The porosity of the test strip of the COVID-19 rapid test kit, as presented in Table 3, is between 9.2 and 20.1%, and in our tested kit, considered to be around 15%. Another critical parameter for simulation is contact angles (wall adhesion), the angle between the fluid and the wall, which can be expected in each iteration in CFD, the so-called dynamic boundary condition.

**Table 3:** Material properties.

Material	Viscosity [cps]	Density [kg/m^3^]	Porosity [%]	Temperature [°C]	Ref.
Bio-sample	0.86–1.5	1035	-	Room temperature, 20	[38,39,40]
Blood	3.5–5.5	1060	-	Body temperature, 37	[40]
Test strip	-	1277	9.2–20.1	Room temperature, 20	[38,41]

**Table 4:** Boundary conditions.

Boundary	Kit	Description	Ref
Inlet	COVID-19 rapid test kit	Velocity inlet	[25,34,42,43]
	Blood glucose kit	Velocity inlet	
	PDMS	Pressure inlet	
Outlet	COVID-19 rapid test kit	Pressure outlet	
	Blood glucose kit		
	PDMS		
Surrounded surface	All	Wall (No-slip)	
Model	COVID-19 rapid test kit	VOF (porous media)	-
	Blood glucose kit	VOF (region register)	
	PDMS	VOF (region register)	
Solution	All	Standard initialization	-
Contact angles	All	Dynamic	-
Time	All	Transient	-
Phases	COVID-19 rapid test kit	Two (air + bio-sample)	-
	Blood glucose kit	Two (air + blood)	
	PDMS	Two (air + blood)	

### 2.3. Governing Equations

To simulate a COVID-19 rapid test kit, the flow movement in the porous zone and porosity of the test strip need to be considered. In Ansys-Fluent, the porous media are modeled by adding a momentum source term to the Navier–Stokes equation as additional forces, Equation (1). The right-hand side of Equation (1) is composed of two parts: a viscous loss term, Darcy (the first term on the right-hand side), and an inertial loss term (the second term on the right-hand side) [44]:

(1) $$S_{i}=-\sum_{j=1}^{3}D_{ij}\mu \upsilon_{j}+\sum_{j=1}^{3}C_{ij}\frac{1}{2}\rho \left|\upsilon \right|\upsilon_{j}$$

where *S_i_*: source term in the *i*th momentum equation (x, y, or z), |*υ*|: velocity magnitude, and *D* and *C* are predefined matrices.

The equation for a uniform porous medium is presented in Equation (2). In Fluent, the user can specify permeability (α) and inertial resistance coefficient (C2) in the porous media setting of Ansys. The defined resistance coefficients will be used in the finite volume discretization of the momentum equation. Fluent iteratively solves the equations and calculates velocity and pressure using numerical methods (SIMPLE, PISO, etc.), incorporating these source terms.

(2) $$S_{i}=-\left(\frac{\mu }{\alpha }\;\upsilon_{i}+C_{2}\frac{1}{2}\rho \left|\upsilon \right|\upsilon_{i}\right)$$

where *α*: permeability, *ρ*: density, *v_i_*: velocity components, *D* and *C*: defined by setting them as diagonal matrices with 1/*α* and *C*_2_ the diagonals, respectively. *C*_2_: inertial resistance factor.

In laminar flows, the pressure drops relate to the velocity, and the constant *C*_2_ can be considered zero. Therefore, the simplified model of the porous media by neglection of the convective acceleration and diffusion in Darcy’s Law is as below:

(3) $$\nabla p=-\frac{\mu }{\alpha }\vec{\upsilon }$$

The pressure changes in the porous region in Ansys-Fluent are as follows:

(4) $$∆p_{x}=\sum_{j=1}^{3}\frac{\mu }{\alpha_{xj}}\upsilon_{j}∆n_{x}$$

(5) $$∆p_{y}=\sum_{j=1}^{3}\frac{\mu }{\alpha_{yj}}\upsilon_{j}∆n_{y}$$

(6) $$∆p_{z}=\sum_{j=1}^{3}\frac{\mu }{\alpha_{zj}}\upsilon_{j}∆n_{z}$$

where *v_j_*: velocity components in *x*, *y*, *z* directions, and Δ*n_x_*, Δ*n_y_*, and Δ*n_z_*: media thicknesses in the *x*, *y*, *z* directions.

To simulate the blood glucose kit and PDMS, the main governing equations (continuity and Navier–Stokes) are as follows [44]:

(7) ∇· u=0

(8) $$\frac{\partial \rho u}{\partial t}+\nabla \cdot (\rho uu)=-\nabla P+\rho g+\nabla \cdot (\mu (\nabla u+\nabla^{T}u))+F_{sf}$$

where *t*: time, *P*: pressure, *μ*: dynamic viscosity, *⍴*: density, *F_sf_*: volumetric surface tension at the interface, and *u*: mixture (bulk) velocity.

The parameter of *F*, which can be between 0 and 1, represents the gas–liquid interface, showing the proportion of the cell volume occupied by the liquid. Therefore, *F* = 1 indicates an entirely filled cell with liquid, and *F* = 0 means a cell filled entirely with gas. The determination of *F* involves solving the continuity equation, assuming incompressibility for the liquid as follows [45]:

(9) $$\frac{\partial F}{\partial t}+u\cdot \nabla F=0$$

The Reynolds number is a dimensionless quantity that helps analyze fluid flow patterns (Laminar conditions *Re* < 2000, turbulent *Re* > 3500). It can be calculated by the following equation:

(10) $$Re=\frac{\rho {UD}_{h}}{\mu }$$

where *⍴* is the density of the fluid, *μ* is the dynamic viscosity of the fluid, *U* is the speed of the flow, and *D_h_* stands for the hydraulic diameter of the channel, which can be calculated by two parameters: *W* that is the width of microchannel, and *H* that is the height of microchannel, as follows:

(11) $$D_{h}=\frac{2WH}{W+H}$$

## 3. Results and Discussion

### 3.1. Experimental and Simulation Results of COVID-19 Rapid Test Kit

**Figure 5:**
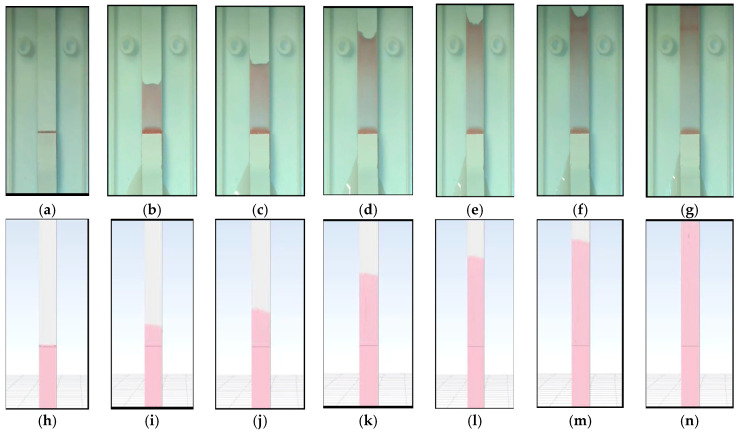
Bio-sample flow in the COVID-19 rapid test kit: (**a**) experimental at second 0; (**b**) experimental at second 10; (**c**) experimental at second 20; (**d**) experimental at second 40; (**e**) experimental at second 50; (**f**) experimental at second 60; (**g**) experimental at second 73; (**h**) CFD result at second 0; (**i**) CFD result at second 10; (**j**) CFD result at second 20; (**k**) CFD result at second 40; (**l**) CFD result at second 50; (**m**) CFD result at second 60; and (**n**) CFD result at second 73.

Analysis of bio-sample flow in the COVID-19 rapid test kit through experimental and CFD simulation is shown in Figure 5, and the total spread time in the length of 20,000 μm was around 73 s.

**Figure 6:**
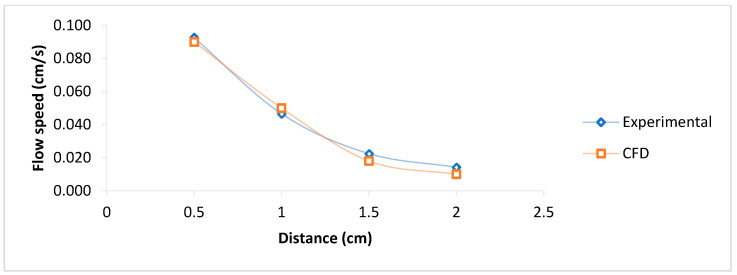
Bio-sample flow validation analysis in the COVID-19 rapid test kit (experimental and CFD).

As can be seen from the experimental analysis, the variations in contact angles (wall adhesion) during the test period mean the necessity of dynamic values for simulation. The bio-sample flow spread speed in the porous zone of the COVID-19 rapid test kits for experimental and CFD simulations is presented in Figure 6, showing good agreement between CFD simulation and experimental results. Moreover, the bio-sample spread simulation within the porous zone determined that the capillary filling speed decreased dramatically in length, as in 20 mm, and it declined nine times, from 0.093 cm/s to 0.014 cm/s. It demonstrates the significance of length optimization in developing these types of kits since it may impact the liquid transit and critical speed for reagent interaction and test results.

### 3.2. Experimental and Simulation Results of the Glucose Kit

**Figure 7:**
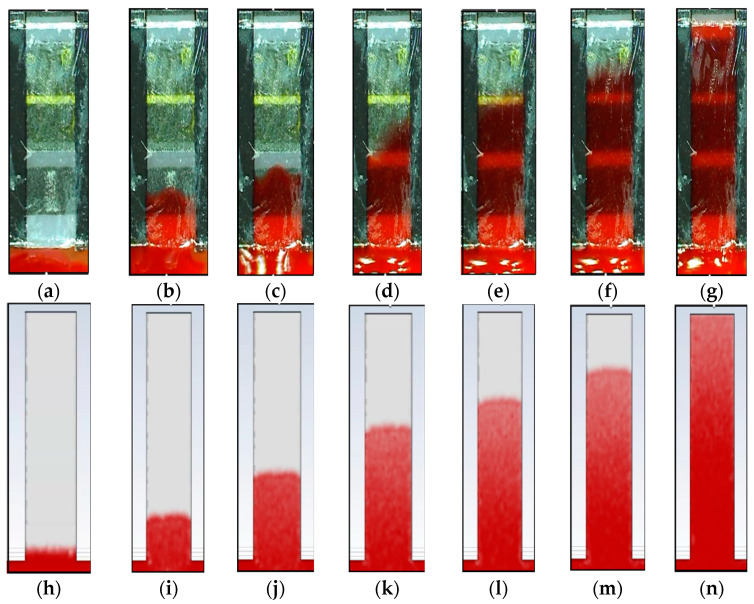
Capillary flow in the glucose kit microchannel: (**a**) experimental at second 0; (**b**) experimental at second 0.1; (**c**) experimental at second 0.2; (**d**) experimental at second 0.3; (**e**) experimental at second 0.4; (**f**) experimental at second 0.5; (**g**) experimental at second 0.7; (**h**) CFD result at second 0; (**i**) CFD result at second 0.1; (**j**) CFD result at second 0.2; (**k**) CFD result at second 0.3; (**l**) CFD result at second 0.4; (**m**) CFD result at second 0.5; and (**n**) CFD result at second 0.7.

The hemodynamic analysis of the blood glucose kit in the experimental and CFD models is shown in Figure 7, and the total movement in the length of 4000 μm was around 0.7 s. The change in contact angle in Figure 7a–g is noticeable, especially between seconds 0.2 (Figure 7c) and 0.3 (Figure 7d), showing that using static contact angles in theoretical equations might not be appropriate. However, in Ansys-Fluent, the values of contact angles can be considered dynamic, which is among the advantages of CFD simulation.

**Figure 8:**
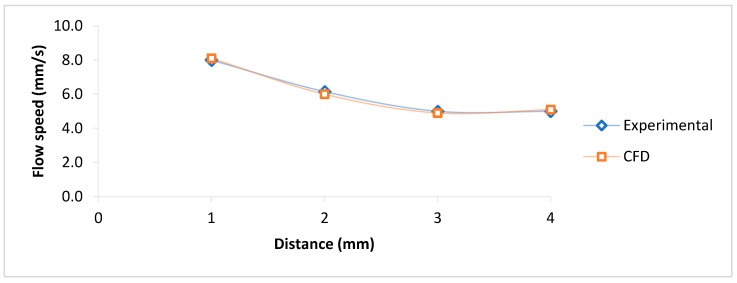
Blood flow velocity validation analysis in the blood glucose kit (experimental and CFD).

The blood velocity in four sectors of the glucose kit (1, 2, 3, and 4 mm) is presented in Figure 8, and the comparisons show good agreement between experimental and CFD simulation. The hemodynamic analysis determined that the capillary filling was decreasing in length, and the decline could depend on the microchannel width and thickness.

### 3.3. Experimental and Simulation Results of PDMS Kit

**Figure 9:**
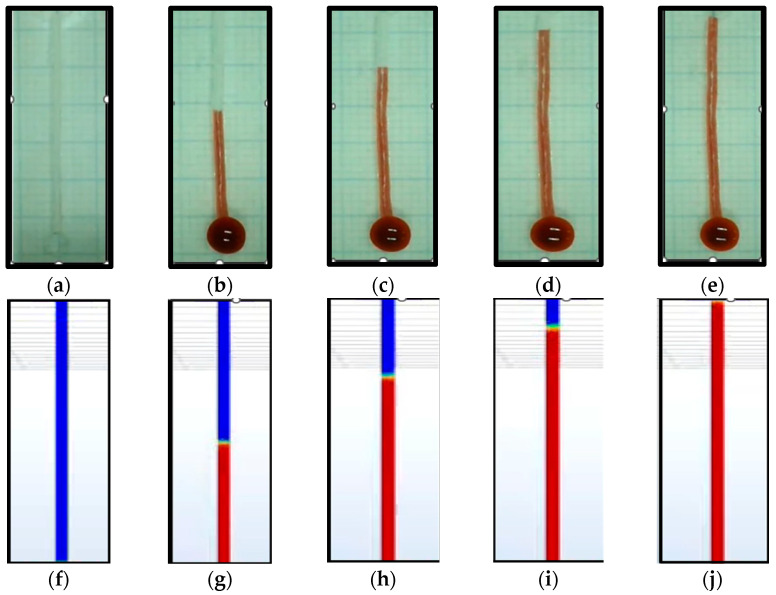
Capillary flow in the PDMS, microchannel diameter of 1000 μm: (**a**) experimental at second 0; (**b**) experimental at second 1; (**c**) experimental at second 2; (**d**) experimental at second 3; (**e**) experimental at second 3.7; (**f**) CFD result at second 0; (**g**) CFD result at second 1; (**h**) CFD result at second 2; (**i**) CFD result at second 3; and (**j**) CFD result at second 3.7.

Hemodynamic analysis of the PDMS kit through experimental and CFD simulations for the microchannel with a diameter of 1000 μm is shown in Figure 9, and the total flow passed in around 3.7 s.

**Figure 10:**
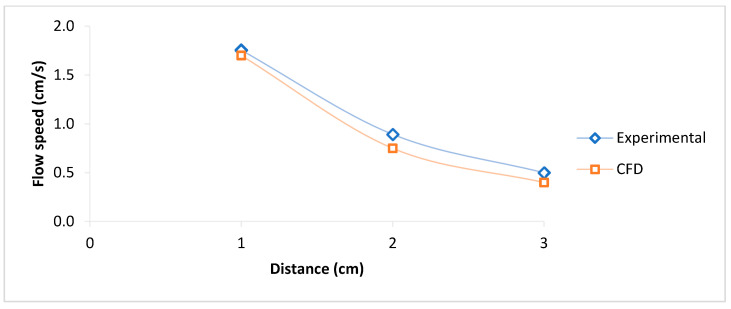
PDMS model validation in the microchannel with a diameter of 1000 μm (experimental and CFD).

The model validation was performed based on the experimental results of the microchannel with a diameter of 1000 μm, as shown in Figure 10, and the validated model was used for the simulation of the other two microchannels.

**Figure 11:**
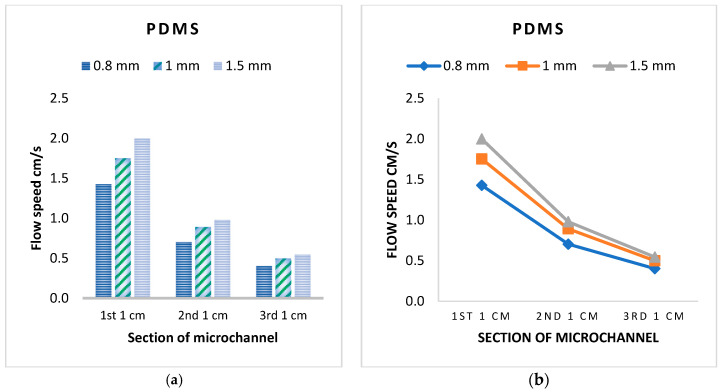
Blood flow velocity comparisons in three different microchannels: (**a**) comparisons in each cm; (**b**) trend in length.

The blood flow velocity in the three microchannels for experimental and CFD simulations is presented in Figure 11. Hemodynamic analysis of the PDMS kit through experimental and CFD simulations showed good agreement between CFD simulation and experimental results. Moreover, the blood speed decreased in length in all three microchannels, which is in agreement with the study of Keshmiri et al. [46], while the blood flow speed in the microchannel with a diameter of 1500 μm was more than the other two microchannels. The average blood speed in the glucose kit was about 0.6 cm/s, more than the PDMS microchannel with the same width, about 1 cm/s. The difference could be due to the thickness of the microchannels, which was 200 μm in the glucose kit and 1000 μm in the PDMS kit.

RMS errors among simulated and experimental results are presented in Table 5, exhibiting low values for all three tests.

The microchannels’ Reynolds numbers are presented in Table 6, demonstrating the variation among 0.4 to 6.4, meaning laminar flow in all sections. In addition, the same speed trend can be seen for Reynolds numbers, which decrease in length.

### 3.4. Challenges, Advantages, and Simplification in Experimental Tests and CFD Simulations

Uncertainties exist in the production, experimental analysis, and numerical results [25,47]. Moreover, more challenges could exist in CFD modeling, such as fluid properties, geometrical development, and grid and mesh properties [48]. A critical point in the simulation of the medical microdevices could be the viscosity of human body fluids, such as blood as a non-Newtonian fluid, which might not be constant [49]. Shi et al. simulated the dynamic behavior of the red blood cell (RBC) deformation within Poiseuille using the finite element model (FEM) and by consideration of the elastic spring network model. The analysis showed that the equilibrium in the shape of RBCs is independent of the initial form of the RBC in the microchannel, and in some cases, the dynamical deformation can be ignored [36]. In addition, the study by Bruus determined that while in the nanoscale, the molecular forces need to be considered, in the micro-scale, the flows can be considered as a continuum as the fluids’ intermolecular distance, which is about 0.3 nm, can be neglected in mm scale of microchannels [17]. In the theoretical calculations of capillary flow, generally, the contact angles are supposed to be static, and the dynamic contact angle is considered insignificant [50], while in CFD simulation with Ansys-Fluent, the angles are adaptive and change dynamically during the simulation, which is among the advantages of CFD simulation in comparison with theoretical results.

The main challenges, uncertainties, and simplifications in the CFD simulation of the selected microdevices are as follows:

- COVID-19 rapid test kit: The flow passage in the plastic bottom of the kit could affect the simulation results. That could be the reason for sharp contact angles, especially in the seconds 50 and 60 in Figure 5.
- Blood glucose kit: As can be seen in the experimental results of the blood glucose kit, the contact angle changes during the capillary movement, which is distinguishable between seconds 0.2 and 0.3 in Figure 7. Therefore, a static or fixed dynamic contact angle can be considered a simplification in the theoretical calculation, while in the CFD model, this problem is solved by adaptive contact angles.
- PDMS: The microdevice is made of PDMS and glass layers. It means two materials with different surface tension coefficients and wall adhesions (contact angles) are simplified to one material in the CFD simulation.

### 3.5. Optimized Dimensions in Microdevices and the Role of CFD Simulations

Optimization of the dimension of microdevices is an important factor as it could affect the test’s speed and achieved results. However, in some cases, such as medical microsensors, it depends not only on the fluid dynamics behavior but also on chemical interactions among liquids (blood, plasma, etc.) and reagents. In fact, the chemical reaction duration could determine the appropriate microfluidic speed, the necessity of laminar or turbulent flow (i.e., mixture of liquids), and other experimental guidelines. In addition, one needs to consider the material properties such as hydrophilic materials, electrode layers, and conductive materials. The main points are reported as below:

- The flow uniformity, which depends largely on the inlet flow rate, shapes of manifolds, and length of microchannels, can be optimized by CFD [18].
- The main parameters that can be optimized include the cross-section, the angles, the length of microchannels, and the ratio of flow rates among samples with sheath flows [51].
- CFD techniques make it possible to analyze microfluidic phenomena that can be difficult to study experimentally in a quicker way, such as heat transfer, velocity fields, shear stresses, and diffusion processes [22].
- ANSYS CFX 2024 can be used to analyze the reaction rate of products to reagents, mixing index (fluid mixing accompanied by chemical reaction), reaction efficiency, and performance index [52].
- Simulation cannot optimize all parts of the sensors. For example, in the COVID-19 test kit, the size of the swab stick for the nasal sample must be designed according to patients’ ease of use, not only CFD modeling [53].

## 4. Conclusions

This research combined experimental and computational fluid dynamics (CFD) to analyze flow behavior in three medical microdevices. Moreover, the study was about the role of computational fluid dynamics (CFD) in simulating medical micro-devices/sensors involving biological fluids, including bio-samples in COVID-19 rapid test kits, blood in blood glucose kits, and PDMS kits. The literature review investigations revealed that blood in the selected experimental analysis could be considered Newtonian fluid, and the RBC deformation would not affect the results. Moreover, in the micro-scale, the liquids can be considered a continuum fluid, and the molecular forces can be neglected as the intermolecular distance, which is about 0.3 nm and is negligible compared to the mm scale of microchannels.

The bio-sample flow spread speed in the porous zone of the COVID-19 rapid test kits for experimental and CFD simulations showed good agreement between CFD simulation and experimental results. Moreover, the bio-sample spread simulation within the porous zone determined that the capillary filling speed decreased dramatically in length, as in just 20mm, it declined nine times, from 0.093 cm/s to 0.014 cm/s. The results show the important role of length optimization in making similar kits, as it could affect the test’s speed and liquid passage for interaction with the reagent to show the test results. The hemodynamic analysis of the blood glucose kit and PDMS kit determined that the capillary filling was decreasing in length, but the speed could depend on the microchannel diameter as the speed was more in the diameter range of 1500 μm than 1000 and 800 μm. CFD results indicated the prominent role of porosity in the simulation of porous media material such as the COVID-19 test kit, as well as surface tension coefficient and wall adhesion (contact angles) in blood glucose kits and PDMS kits. In addition, the flow speed would depend on the thickness of the microchannel as the average blood speed in the glucose kit was about 0.6 cm/s, less than the PDMS microchannel with the same width, with an average speed of 1 cm/s. The difference could be due to the thickness variation that in the glucose kit is 200 μm and in the PDMS about 1mm. Reynolds number analysis showed that the blood glucose kit ranges between 0.4 and 0.7, which was highest at the beginning and decreased in length. In PDMS kits, the Reynolds’ number ranged between 0.9 and 6.4, and there was an increase in the channel with a higher width.

Comparisons among simulation and experimental results revealed the accuracy of CFD simulation, showing the general errors in predicting the flow speed by theoretical equations due to the changes in wall adhesion (contact angles) during the capillary flow, which adaptive contact angles could overcome in numerical simulation. In fact, adaptive dynamic contact angles in CFD simulation by Ansys-Fluent could result in more accurate prediction for sensor optimization than simplified theoretical techniques. In conclusion, validated CFD models could be valuable tools in simulating human body fluids within medical microdevices, resulting in faster sensor optimization and development.

The results showed the impact of kit length and thickness on the flow speed, but the maximum/minimum/optimized dimensions were not among the aims of this research and are recommended for future studies. Moreover, the role of multi-materials in the test kits was another uncertainty of the current study, and further analysis is suggested for future research.

## Figures and Tables

**Table 1:** Experimental errors in the three case studies.

Item	COVID-19 Rapid Test Kit	Blood Glucose Kit	PDMS
Number of Tests	3	4	3
Standard Deviation	3	0.001	0.04
Error %	1.37%	0.04%	0.36%

**Table 2:** Mesh details of the three case studies.

Mesh	COVID-19 Rapid Test Kit	Blood Glucose Kit	PDMS
Number of elements	3,000,000	301,050	678,296
Number of nodes	3,251,332	436,624	458,527
Element size	20 μm	50 μm	300 μm

**Table 5:** RMS error among simulated and experimental results.

Material	COVID-19 Rapid Test Kit	Blood Glucose Kit	PDMS
Root mean square error (RMSE)	0.004	0.116	0.105

**Table 6:** Reynolds number in blood glucose kit and PDMS.

Microdevice		Distance	Re
Blood glucose kit		1st mm	0.7
		2nd mm	0.5
		3rd mm	0.4
		4th mm	0.4
PDMS	Microchannel with a width of 800 μm	1st cm	3.4
		2nd cm	1.7
		3rd cm	0.9
	Microchannel with a width of 1000 μm	1st cm	4.6
		2nd cm	2.4
		3rd cm	1.3
	Microchannel with a width of 1500 μm	1st cm	6.4
		2nd cm	3.1
		3rd cm	1.7

## Data Availability

Data will be made available on request.

## Abbreviations

The following abbreviations are used in this manuscript:

CFD	Computational Fluid Dynamics
COVID-19	Corona Virus Disease 2019
DPD	Dissipative particle dynamics method
DSMC	Direct Simulation Monte Carlo
FD	Flow Diverter
FEM	Finite Element Model
FPC	Frame per second
LBM	Lattice Boltzmann methods
LMTF	a liquid membrane in Taylor flow
LoC	Lab-on-a-Chip
MD	Molecular Dynamics
PDMS	Polydimethylsiloxane
RBC	Red Blood Cell
VOF	Volume of fluids

## References

[1] Li S., Ma Z., Cao Z., Pan L., Shi Y. (2020). Advanced Wearable Microfluidic Sensors for Healthcare Monitoring. Small.

[2] Takken M., Wille R. (2022). Simulation of Pressure-Driven and Channel-Based Microfluidics on Different Abstract Levels: A Case Study. Sensors.

[3] Su W., Cook B.S., Fang Y., Tentzeris M.M. (2016). Fully inkjet-printed microfluidics: A solution to low-cost rapid three-dimensional microfluidics fabrication with numerous electrical and sensing applications. Sci. Rep..

[4] Sugiyama H., Tsunemitsu K., Onoe H., Obata K., Sugioka K., Terakawa M. (2021). Microfabrication of cellulose nanofiber-reinforced hydrogel by multiphoton polymerization. Sci. Rep..

[5] Pradeep A., Raveendran J., Babu T.G.S. (2022). Design, fabrication and assembly of lab-on-a-chip and its uses. Prog. Mol. Biol. Transl. Sci..

[6] Podunavac I., Djocos M., Vejin M., Birgermajer S., Pavlovic Z., Kojic S., Petrovic B., Radonic V. (2023). 3D-Printed Microfluidic Chip for Real-Time Glucose Monitoring in Liquid Analytes. Micromachines.

[7] Ha N.S., De Raad M., Han L.Z., Golini A., Petzold C.J., Northen T.R. (2021). Faster, better, and cheaper: Harnessing microfluidics and mass spectrometry for biotechnology. RSC Chem. Biol..

[8] Fink G., Ebner P., Hamidović M., Haselmayr W., Wille R. Accurate and Efficient Simulation of Microfluidic Networks. Proceedings of the 26th Asia and South Pacific Design Automation Conference.

[9] Xie Z., Pu H., Sun D.W. (2021). Computer simulation of submicron fluid flows in microfluidic chips and their applications in food analysis. Compr. Rev. Food Sci. Food Saf..

[10] Lambert B., Van De Wiele C. (2005). Treatment of Hepatocellular Carcinoma by Means of Radiopharmaceuticals.

[11] Stone H.A., Stroock A.D., Ajdari A. (2004). Engineering flows in small devices: Microfluidics toward a lab-on-a-chip. Annu. Rev. Fluid Mech..

[12] Zhong S., Xue L., Wang Y., Zhang C., Liu N., Li L., Zhang Q., Yue T. (2024). Paper-based microfluidic chips for wide time range fluid control based on knife crafting and laser cutting. Sens. Actuators B Chem..

[13] Gad-el-Hak M. (2001). Flow physics in MEMS. Mec. Ind..

[14] Kowalewski W., Roszak M., Kolodziejczak B., Ren-Kurc A., Brȩborowicz A. (2016). Computational fluid dynamics methods and their applications in medical science. Stud. Log. Gramm. Rhetor..

[15] Li Q., Luo K.H., Kang Q.J., He Y.L., Chen Q., Liu Q. (2016). Lattice Boltzmann methods for multiphase flow and phase-change heat transfer. Prog. Energy Combust. Sci..

[16] Chen S., Doolen G.D. (1998). Lattice boltzmann method for fluid flows. Annu. Rev. Fluid Mech..

[17] Goetting-Minesky M.P., Mullin B.C. (1994). Differential gene expression in an actinorhizal symbiosis: Evidence for a nodule-specific cysteine proteinase. Proc. Natl. Acad. Sci. USA.

[18] Tonomura O., Tanaka S., Noda M., Kano M., Hasebe S., Hashimoto I. (2004). CFD-based optimal design of manifold in plate-fin microdevices. Chem. Eng. J..

[19] Zhang M., Zheng A., Zheng Z.C., Wang M.Z. (2019). Multiphase flow experiment and simulation for cells-on-a-chip devices. Proc. Inst. Mech. Eng. Part H J. Eng. Med..

[20] Hodgson G., Passmore M., Skarysz M., Garmory A., Paolillo F. (2021). Contact angle measurements for automotive exterior water management. Exp. Fluids.

[21] Xue L., Guo C., Zhang Y., Xu Y., Li B. (2022). Parametrical Study on the Capillary Flowing Characteristics of the Parallel Microchannel Array. Crystals.

[22] Carvalho V., Rodrigues R.O., Lima R.A., Teixeira S. (2021). Computational simulations in advanced microfluidic devices: A review. Micromachines.

[23] Ahmed F., Yoshida Y., Wang J., Sakai K., Kiwa T. (2021). Design and validation of microfluidic parameters of a microfluidic chip using fluid dynamics. AIP Adv..

[24] Llano-Serna C.E., Fernandes A.C., Krühne U., Fontalvo J., Prado-Rubio O.A. (2021). Computational fluid dynamics (CFD) assisted design and prototyping of the immiscible drop separation section for an intensified perstraction system. Chem. Eng. Process. Process. Intensif..

[25] García B.F., Mousaviraad M., Saraji S. (2022). Verification and validation for microfluidic CFD simulations of Newtonian and non-Newtonian flows. Appl. Math. Model..

[26] Frolov S.V., Sindeev S.V., Kirschke J.S., Arnold P., Prothmann S., Liepsch D., Balasso A., Potlov A., Larrabide I., Kaczmarz S. (2018). CFD and MRI studies of hemodynamic changes after flow diverter implantation in a patient-specific model of the cerebral artery. Exp. Fluids.

[27] Cito S., Pallares J., Fabregat A., Katakis I. (2012). Numerical simulation of wall mass transfer rates in capillary-driven flow in microchannels. Int. Commun. Heat Mass Transf..

[28] Schönfeld F., Hardt S. (2009). Dynamic contact angles in CFD simulations. Comput. Fluids.

[29] Arabghahestani M., Poozesh S., Akafuah N.K. (2019). Advances in computational fluid mechanics in cellular flow manipulation: A review. Appl. Sci..

[30] Pirouz B., Mazzeo D., Palermo S.A., Naghib S.N., Turco M., Piro P. (2021). Cfd investigation of vehicle’s ventilation systems and analysis of ach in typical airplanes, cars, and buses. Sustainbility.

[31] Pirouz B., Piro P. (2025). Analysis of Computational Fluid Dynamics Approaches for the Development of Microfluidic Devices. Lecture Notes in Computer Science (Including Subseries Lecture Notes in Artificial Intelligence and Lecture Notes in Bioinformatics).

[32] Nagler O., Trost M., Hillerich B., Kozlowski F. (1998). Efficient design and optimization of MEMS by integrating commercial simulation tools. Sens. Actuators A Phys..

[33] O’Connor J., Day P., Mandal P., Revell A. (2016). Computational fluid dynamics in the microcirculation and microfluidics: What role can the lattice Boltzmann method play?. Integr. Biol..

[34] Oliveira M.S.N., Alves M.A., Pinho F.T., McKinley G.H. (2007). Viscous flow through microfabricated hyperbolic contractions. Exp. Fluids.

[35] Peng S., Chen Q., Liu E. (2020). The role of computational fluid dynamics tools on investigation of pathogen transmission: Prevention and control. Sci. Total Environ..

[36] Shi L., Pan T.W., Glowinski R. (2014). Three-dimensional numerical simulation of red blood cell motion in Poiseuille flows. Int. J. Numer. Methods Fluids.

[37] Liu H., Lan L., Abrigo J., Ip H.L., Soo Y., Zheng D., Wong K.S., Wang D., Shi L., Leung T.W. (2021). Comparison of Newtonian and Non-newtonian Fluid Models in Blood Flow Simulation in Patients With Intracranial Arterial Stenosis. Front. Physiol..

[38] Jordan M., Švarc T., Majerič P., Rudolf R., Zadravec M. (2023). Reconstruction of a Fluid Bed Device for Separating Granular Material from the Grinding Process of Rapid Antigen Tests. Processes.

[39] Sarkar A., Xu F., Lee S. (2019). Human saliva and model saliva at bulk to adsorbed phases—Similarities and differences. Adv. Colloid Interface Sci..

[40] Nader E., Skinner S., Romana M., Fort R., Lemonne N., Guillot N., Gauthier A., Antoine-Jonville S., Renoux C., Hardy-Dessources M.D. (2019). Blood rheology: Key parameters, impact on blood flow, role in sickle cell disease and effects of exercise. Front. Physiol..

[41] Astuti N.H., Wibowo N.A., Ayub M.R.S.S.N. (2018). The Porosity Calculation of Various Types of Paper Using Image Analysis. J. Pendidik. Fis. Indones..

[42] Gupta R., Fletcher D.F., Haynes B.S. (2009). On the CFD modelling of Taylor flow in microchannels. Chem. Eng. Sci..

[43] Aladese A.D., Jeong H.-H. (2022). Numerical and experimental investigations of uniform fluid distribution for droplet formation in parallelized microfluidics. Front. Sens..

[44] ANSYS FLUENT ANSYS Fluent 12.0 User’s Guide. https://www.afs.enea.it/project/neptunius/docs/fluent/html/ug/main_pre.htm.

[45] Saha A.A., Mitra S.K., Tweedie M., Roy S., McLaughlin J. (2009). Experimental and numerical investigation of capillary flow in SU8 and PDMS microchannels with integrated pillars. Microfluid. Nanofluidics.

[46] Keshmiri K., Huang H., Jemere A.B., Nazemifard N. (2020). Investigation of Capillary Filling Dynamics of Multicomponent Fluids in Straight and Periodically Constricted Microchannels. Langmuir.

[47] Silvestri L., Saraceni M., Bongioannini Cerlini P. (2022). Quality management system and design of an integrated mesoscale meteorological network in Central Italy. Meteorol. Appl..

[48] Pereira J.M.C., Serra e Moura J.P., Ervilha A.R., Pereira J.C.F. (2013). On the uncertainty quantification of blood flow viscosity models. Chem. Eng. Sci..

[49] Pavlin-Premrl D., Boopathy S.R., Nemes A., Mohammadzadeh M., Monajemi S., Ko B.S., Campbell B.C.V. (2021). Computational Fluid Dynamics in Intracranial Atherosclerosis—Lessons from Cardiology: A Review of CFD in Intracranial Atherosclerosis. J. Stroke Cerebrovasc. Dis..

[50] Yang D., Krasowska M., Priest C., Popescu M.N., Ralston J. (2011). Dynamics of capillary-driven flow in open microchannels. J. Phys. Chem. C.

[51] Liu X., Zhou J., Yan R., Tang T., Wei S., Li R., Hou D., Weng Y., Wang D., Shen H. (2023). An optimized PDMS microfluidic device for ultra-fast and high-throughput imaging flow cytometry. Lab Chip.

[52] Santana H.S., da Silva A.G.P., Lopes M.G.M., Rodrigues A.C., Taranto O.P., Lameu Silva J. (2020). Computational methodology for the development of microdevices and microreactors with ANSYS CFX. MethodsX.

[53] Kumwenda M.K., Mukoka M., Reipold-Ivanova E., Mhango O., Dunkley Y., Abok F., Sibanda E., Watadzaushe C., Corbett E.L., Choko A.T. (2024). Optimising instructional materials for Covid19 rapid tests for self-sampling and testing: Mapping the optimization process of manufacturer’s instructions for use for selftesting RDTs intended for low-literacy contexts. PLoS ONE.

